# Irritability through Research Domain Criteria: an opportunity for transdiagnostic conceptualisation

**DOI:** 10.1192/bjo.2020.168

**Published:** 2021-01-19

**Authors:** Erica Bell, Richard A. Bryant, Philip Boyce, Richard J. Porter, Gin S. Malhi

**Affiliations:** Faculty of Medicine and Health, University of Sydney, Australia; School of Psychology, University of New South Wales, Australia; Discipline of Psychiatry, Sydney Medical School, Westmead Clinical School, University of Sydney, Australia; and Perinatal Psychiatry Clinical Research Unit, Westmead Hospital, Australia; Department of Psychological Medicine, University of Otago, New Zealand; Department of Psychiatry, University of Sydney, Australia

**Keywords:** Irritability, Research Domain Criteria, children and adolescents, mood disorders, phenomenology

## Abstract

Irritability is a transdiagnostic phenomenon that, despite its ubiquity and significant impact, is poorly conceptualised, defined and measured. As it lacks specificity, efforts to examine irritability in adults by using a diagnostic category perspective have been hamstrung. Therefore, using a Research Domain Criteria (RDoC) approach to examine irritability in adults, which spans many constructs and domains, may have a better chance of yielding underlying mechanisms that can then be mapped onto various diagnostic categories. Recently, a model has been proposed for irritability in children and adolescents that uses the RDoC framework; however, this model, which accounts for chronic, persistent irritability, may not necessarily transpose to adults. Therefore, use of the RDoC framework to examine irritability in adults is urgently needed, as it may shed light on this currently amorphous phenomenon and the many disorders within which it operates.

The long-awaited update of the DSM in 2013 was upstaged somewhat by the concurrent release of the Research Domain Criteria (RDoC), which declared a divergence in research direction. Although the two approaches were eventually presented as being synergistic, they clearly propose alternative approaches (a top-down perspective is adopted by DSM versus the largely bottom-up approach championed by the RDoC). Interestingly, it has since become evident that these two ‘methodologies’ are not only synergistic, but in fact, interdependent. This is because diagnostic categories created by the DSM are needed to draw samples from clinical practice to make effective use of the RDoC framework. Reciprocally, to understand the substrates of disorders, the RDoC approach provides a logical matrix to examine the correlates of DSM categories, to understand the substrates of disorders. Nevertheless, jointly, the two systems provide an advance that is evident with some phenomena more so than others. For instance, irritability, which seems to be transdiagnostic in nature, lends itself to inquiry through RDoC. Thus, in this editorial, we consider how examining irritability through the RDoC lens may provide a deeper understanding of a ‘symptom’ that has proven to be remarkably difficult to define – one might even say, irritatingly so.

## Irritability

In psychiatry, eliciting key diagnostic symptoms can be difficult because some symptoms, such as delusional mood (*whanstimmung*) are relatively uncommon, and thus inherently challenging to define. In contrast, irritability is commonplace and very familiar. Indeed, it is a phenomenon that everyone has experienced at some time or another. However, despite this, it is loosely conceptualised, lacks a validated definition and is consequently poorly measured, both quantitatively and qualitatively.^1^ At the same time, it is clearly important clinically, as it features seemingly ubiquitously in psychiatric psychopathology across the age spectrum, traversing theoretical diagnostic borders of disorder type. Although irritability causes significant distress and impairment, and is associated with poorer outcomes, it is not effectively addressed or specifically targeted with current treatments. Therefore, irritability is an optimal candidate for examination through the RDoC framework, as it is transdiagnostic and widely experienced, and by understanding it as a phenomenon, effective interventions can be developed to improve outcomes and prognosis.

## Irritability in children and adolescents and disruptive mood dysregulation disorder

In recent years, the significance of irritability has been increasingly recognised because of its associated impairment, especially in children and adolescents. In youth, it is deemed to be the core feature of disruptive mood dysregulation disorder (DMDD). Although the diagnosis of DMDD itself is highly controversial and contested by many, the centrality and impact of irritability in children and adolescents is widely acknowledged. This is because in practice, it is often the primary complaint of patients (and parents) when presenting to a psychiatrist.^2^ Hence, the conceptualisation of irritability and its underlying mechanisms has been the focus of many recent studies, and increasingly, the RDoC framework has been employed to map irritability in childhood psychiatric disorders, including DMDD.

**Fig. 1:**
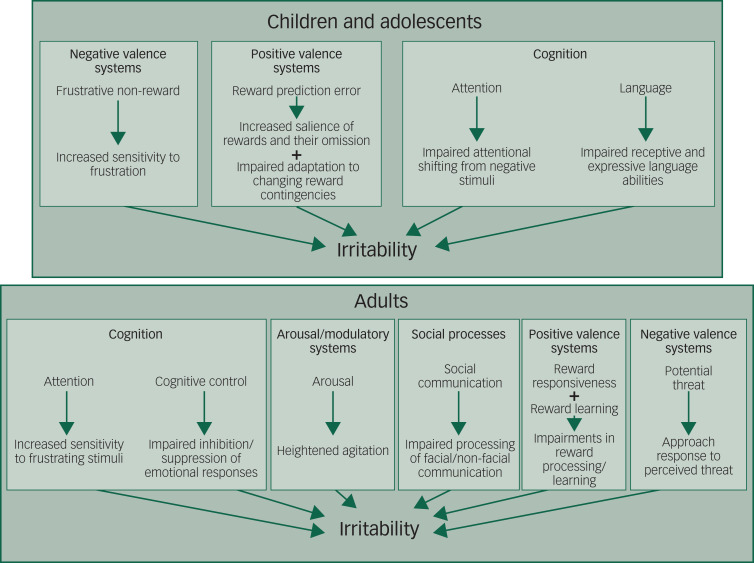
Comparison of Research Domain Criteria (RDoC) domains used to conceptualise irritability in children and adolescents versus adults. This schematic illustrates the RDoC domains and its constructs that have been proposed in child and adolescent models of irritability. In adults, the domains and constructs are proposed based on the current conceptualisation of irritability as a ‘state of increased agitation and sensitivity to sensory stimuli accompanied by a lowered threshold for angry/aggressive responses to stimuli’.

Currently, DMDD has been conceptualised within the RDoC framework as comprising dysfunction of frustrative non-reward (negative valence domain), errors in reward prediction (positive valence domain) and impairments in attention and language (cognitive domain)^3^ (see Fig. 1). Irritable children, such as those with DMDD, have demonstrated an increased sensitivity to frustration in the absence of reward. This can result in behavioural consequences, such as aggression and anger, when rewards are withheld. In addition, stimuli indicating rewards and their omission have been found to be more salient among irritable children, and their ability to adapt their behaviour to changing reward contingencies may also be impaired. Together, these findings indicate that irritable youth are more sensitive to rewards and their omission, but are impaired in their ability to adjust their behaviour to attain them. This increases their likelihood of experiencing frustration, to which they are more sensitive, and so their response is likely to be ‘exaggerated’, resulting in anger or aggression. Further, irritable children have been proposed to be impaired in their ability to engage strategies to regulate their emotional responses with attentional shifting and language. In other words, once the individual becomes frustrated, they are then impaired in their ability to regulate their emotional responses.

These components have been theorised as the driving underlying processes that lead to chronic or persistent irritability with temper/behavioural outbursts, and are based on neuroimaging and behavioural data. However, it is important to bear in mind that the RDoC components that have been postulated to underlie irritability in children and adolescents may not meaningfully inform irritability observed in adults.

## Irritability in adults

Irritability has attracted less research interest in adult psychiatric disorders than in children and adolescents. This is puzzling, given that irritability is a core diagnostic feature of many psychiatric disorders (mood, anxiety, substance use and addiction, personality disorders and trauma- and stress-related disorders) and a supportive diagnostic feature in several others. Nevertheless, despite its ubiquity and diagnostic salience, the transdiagnostic nature of irritability may be its biggest drawback to research investigating its underlying mechanisms, as it also means it lacks specificity. Further, its potential overlap with non-psychiatric states has been recognised as problematic because it may contribute to overpathologising normative states. For example, irritability was replaced in post-traumatic stress disorder criteria in the DSM-5 with anger outbursts to more accurately identify non-normative states.^4^

As irritability permeates diagnostic boundaries, research approaching irritability from the diagnostic category perspective has failed to produce a consensus on how best to define it, especially in the context of adult psychopathology.^1^ Persistent and chronic irritability is less common among adults than in children and adolescents. Instead, it is often episodic, occurs in the context of episodes of psychiatric illness and is state-dependent.

In this context, irritability can be conceptualised as the following: a subjective experience of increased agitation and sensitivity to sensory stimuli, characterised by a non-cognitively mediated lowered threshold for responding to stimuli with anger or aggression.^1^ Critically, this conceptualisation of irritability is primarily that of a subjective experience that may not necessarily result in an observable behaviour. This is critical because, in children and adolescents, the primary method to measure irritability is through observations of externalising behaviours, such as temper outbursts or caregiver reports. Therefore, examining irritability in adults where subjective experience can be more easily measured, may provide valuable refinement to the current child and adolescent model of irritability utilising the transdiagnostic RDoC framework.

## Potential domains and constructs

Irritability could be examined across several RDoC domains: cognitive systems (attention and cognitive control), arousal and regulatory systems, social processes, and positive and negative valence systems. Several of these constructs overlap with those currently acknowledged in the child and adolescent model (see Fig. 1); however, there are some key examples that may nuance and expand upon this model in adults that could be explored.

Although impaired language capabilities may address emotion regulation deficits in children and adolescents, this does not necessarily apply to adults, where language is assumed to be fully developed. Instead, in adults, irritability may be explored through the constructs of cognitive control, specifically the inhibition/suppression of responses. As irritability in adults is proposed to entail a lowered threshold for angry/aggressive responses, impairments in mediating behavioural responses to negative stimuli may be apparent. There is some preliminary evidence for this, as neurobiological examinations have shown that negative stimuli increased activation in the amygdala and decreased activity in the medial orbitofrontal cortex in symptomatic women with premenstrual dysphoric disorder.^5^ These brain regions are implicated in emotional responsivity to emotional stimuli and the mediation of behavioural responses, accordingly. This indicates that irritability may involve impairments in cognitive control of emotional and behavioural responses, and therefore could be explored through the RDoC domain of the cognition.

In addition to the domain of cognitive control, impairments in the domain of social processes may be particularly relevant to the emergence of irritability in adults. As sensitivity to stimuli and the likelihood of reacting with anger or aggression are thought to be increased during periods of irritability, processing of specific stimuli, such as facial and non-facial communication, may be affected. Impairments in the processing of facial emotion have been found in irritable youth^6^ and, in adults, hostile attribution biases to social information processing were associated with impulsive aggression, which is closely related to irritability.^7^ Clearly, the relationship between the construct of social processes, in particular facial and non-facial communication, and irritability requires further examination. This is particularly important because findings in this domain may disentangle the distinct contributions of irritability and underlying psychiatric disorders to deficits in social information processing known to occur throughout various psychiatric disorders, such as bipolar disorder.

The constructs of reward responsiveness and reward learning have been implicated in the child and adolescent irritability model, but they may also inform adult presentations of irritability, particularly in the context of mood disorders. As sensitivity to sensory stimuli is increased and the threshold for response to stimuli is decreased during irritability, it may be that the perceived salience of rewards and/or their omission is atypical in adults. In mood disorders, reward processing is impaired: patients with depression show deficits in processing of reward stimuli, and patients experiencing mania show impaired prediction error coding when a reward is omitted, implying deficits in reward learning.^8,9^ As irritability is a core feature of mania and mixed depression, exploration of reward responsiveness and reward learning may have significant implications for our understanding of how the presence of irritability affects these constructs in mood disorders, and in psychopathology more broadly.

A key aspect of irritability that is currently not captured in the existing RDoC framework is the approach response to threatening or negative emotional stimuli that has been found in both youth and adults.^10,11^ This is of interest as irritability is typically conceptualised as a ‘negative’ state or experience, conceptualised within the negative valence systems domain of RDoC in youth. However, irritability appears to evoke an opposite response to the typical avoidance of threatening stimuli, as seen in the context of anxiety. In adults, irritability has been closely associated with anxiety, with high rates of co-occurrence and relationships with other anxiety symptoms.^12^ Irritability has similar neurobiological findings to anxiety, with increased monoamine transmission^13,14^ and overlapping patterns of activity across key brain areas, such as the central noradrenergic system, the amygdala and its connection to the frontal cortices.^15^ Therefore, it has been proposed that irritability and anxiety reflect two sides of the approach/avoidance response to stimuli, and as such, exploration of a new negative valence construct that entails an approach response to threatening/negative stimuli should be investigated as a potential way to map irritability on to neurobiological processes.

## Future

Currently, the examination of irritability in adults, and in particular in the variety of psychiatric disorders within which irritability operates, is both scarce and difficult to unify. Rather than examining irritability through the context of individual disorders or groups of disorders, by adopting a transdiagnostic perspective, research may begin to unravel the nature of this common and perplexing phenomenon. There exists significant potential to understand the underlying mechanisms of irritability in adult psychiatric disorders by utilising the RDoC framework. This perspective may yield significant insights that allow for an accurate conceptualisation of irritability, which may also reveal information that can be used to refine our conceptualisation and categorisation of psychiatric disorders more broadly. Importantly, investigating the mechanisms that underlie irritability will inform our understanding of both this phenomenon, and the many disorders within which it manifests. Doing so provides an opportunity to develop effective treatments that may be able to target the underpinning processes that drive irritability, thus improving outcomes for individuals with a wide range of psychiatric disorders.

## Supplementary material

For supplementary material accompanying this paper visit http://dx.doi.org/10.1192/bjo.2020.168. click here to view supplementary material
