# Supplementary material for: Irritability through Research Domain Criteria: an opportunity for transdiagnostic conceptualisation
Source: BJPsych Open. 2021 Jan 19;7(1):e36. doi: 10.1192/bjo.2020.168 (PMC8058909; doi:10.1192/bjo.2020.168)
Supplement: Supplementary file 1 [file S2056472420001684sup001.docx]

**Author biographical details:**

Erica Bell is a Doctoral student in the Faculty of Medicine and Health at the University of Sydney, Australia.

Richard Bryant is a Scientia Professor and NHMRC Senior Principal Research Fellow, School of Psychology, University of New South Wales, Australia.

Philip Boyce is Professor of Psychiatry at the University of Sydney and Head of the Perinatal Psychiatry Clinical Research Unit at Westmead Hospital.

Professor Richard Porter is head of the Department of Psychological Medicine, University of Otago, Christchurch. His research is primarily in psychological treatments for mood disorders.

Gin S. Malhi is a Professor and Chair of the Discipline of Psychiatry at the University of Sydney, Australia.
